# Unplanned Excision of Extremity Soft Tissue Sarcoma in Korea: A Nationwide Study Based on a Claims Registry

**DOI:** 10.1371/journal.pone.0134354

**Published:** 2015-08-03

**Authors:** Seungcheol Kang, Han-Soo Kim, Ilkyu Han

**Affiliations:** 1 Department of Orthopaedic Surgery, Seoul National University Hospital, Seoul, Korea; 2 Musculoskeletal Tumor Center, Seoul National University Cancer Hospital, Seoul, Korea; Harvard Medical School; Massachusetts General Hospital, UNITED STATES

## Abstract

Unplanned excision of extremity soft tissue sarcoma (STS) is common and has detrimental effects not only on patients’ oncologic outcomes but also on functional and economic issues. However, no study has analyzed a nationwide population-based database. To estimate the incidence and treatment pattern of unplanned excision in extremity STS in the Korean population, a nationwide epidemiologic study was performed using the Korean Health Insurance Review and Assessment Service database, a centralized nationwide healthcare claims registry of Korea that covers the entire Korean population. Among 1,517 patients with extremity STS in the 4-year study period, 553 (36.5%) underwent unplanned excision (unplanned group). About 80% of unplanned excisions were performed in tertiary or general hospitals. Of the unplanned group, 240 (43.4%) underwent re-excision with or without radiation therapy and/or chemotherapy, and 51 (9.2%) did not undergo re-excision but were treated with radiation therapy and/or chemotherapy; whereas, 262 (47.4%) did not undergo any further treatment following unplanned excision. This study is the first nationwide population-based study on the unplanned excision of extremity STS. The results may have implications in establishing preventive or therapeutic measures to reduce the burden of unplanned excision of extremity STS.

## Introduction

Soft tissue sarcoma (STS) of the extremities is a rare malignant tumor developing from the mesoderm and constitutes <0.6% of all malignant tumors [1,2]. On the other hand, benign soft tissue tumors of the extremities are much more common, with 100–300 times the incidence of STS. Extremity STS is often mistaken for benign tumors; initial resection is performed as if they were benign tumors [3,4]. Unplanned excision of an STS is defined as the operation performed for the gross removal of an STS without regard for preoperative imaging or the necessity to remove a margin of normal tissue covering the cancer [5–7].

In cases of unplanned excision, it is generally assumed that re-excision with a wide margin of the normal surrounding tissues is required to treat gross or microscopic tumors; this may necessitate multiple surgeries and prolonged hospital stay. Thus, unplanned excision has a detrimental effect not only on patients’ oncologic outcomes but also on functional and economic aspects. Estimating the burden of unplanned excision of STS is necessary to understand its impact on public health. However, studies on unplanned excision of STS involve hospital-based settings [7–9], and no study has been performed on a nationwide population-based database.

South Korea operates a mandatory nationwide healthcare system with a centralized healthcare claims database: the Korean Health Insurance Review and Assessment Service (HIRA) database. The HIRA database contains all medical and prescription drug claim records of the entire South Korean population and provides a unique nationwide information source on healthcare resource utilization [10–12]. In this study, we utilized the HIRA database to estimate the incidence and treatment patterns of unplanned excision in extremity STS in the Korean population.

## Materials and Methods

### Data source

The Korean National Health Insurance (NHI) is mandatory social insurance and the only public medical insurance program run by the Korean government [10–12]; approximately 97% of the Korean population is covered by the NHI. Cancer patients insured by the NHI pay only a 5% copayment, and hospitals are required to submit claims for the remaining 95% of expenses for both inpatient and outpatient care, for which a histologic diagnosis of a cancer is required. The submitted claims encompass data on diagnoses, procedures, prescription records, demographic information, and direct medical costs. The remaining 3% of the population uninsured by the NHI is covered by the Medical Aid Program. Claims data submitted by the Medical Aid Program are also reviewed by HIRA. Therefore, virtually all claims data of the entire Korean population are registered in the HIRA database. The HIRA database was established on the basis of the 6^th^ edition of Korean Classification of Disease (KCD-6), which is the modified version of the International Classification of Disease, 10^th^ revision (ICD-10), for the Korean healthcare system. The HIRA database has been used for many epidemiological studies [13–15].

All Korean residents receive a unique identification number at birth, called a Korean Resident Registration Number, which enables the identification of every citizen. Korean Resident Registration Numbers are widely used in government programs including the NHI and the HIRA database. Therefore, the HIRA database can be used to obtain the healthcare records and demographic characteristics of specific patients without any duplications or omissions. However, public access to the HIRA database is prohibited through legislation on Personal Information Protection of Public Institutions. Restricted access is allowed with permission from the Deliberative Committee of HIRA. Accordingly, the present study was deemed as in conformity with the common good and was subsequently approved by the Deliberative Committee of HIRA. The HIRA provided only de-identified data within the last 5 years. The following information of each de-identified individual was provided: (1) diagnostic codes and their claim dates, referral level of the hospital, and regional distribution; and (2) procedure codes and their claim dates, referral level of the hospital, and regional distribution among the patients with claim records of a diagnosis of STS. Ethical approval for this study was exempted by the Seoul Nation University Hospital Institutional Review Board because the authors only accessed data that had already been de-identified. The data can be requested at the HIRA website: http://www.hira.or.kr/dummy.do?pgmid=HIRAA070001000430.

### Study population

**Fig 1:**
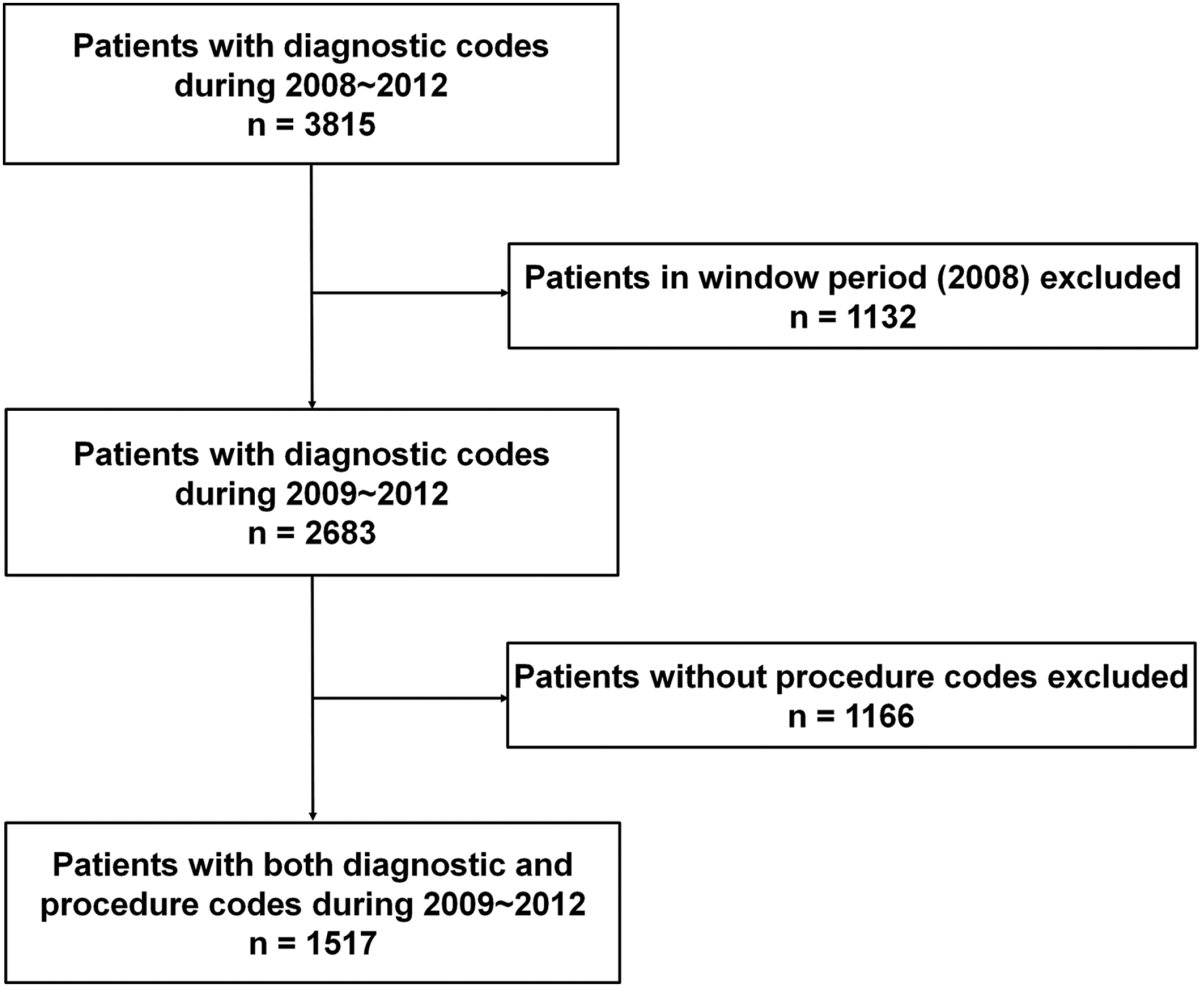
Flowchart for the selection of patients with extremity soft tissue sarcoma.

To identify extremity STS patients in the entire Korean population from the HIRA database, all claims records of outpatient visits or hospital admissions of patients with extremity STS from January 1, 2008 to December 31, 2012 were searched. All extremity STS patients with both diagnostic and procedural codes were included (Fig 1). The diagnostic codes of extremity STS included “malignant neoplasm of peripheral nerves and autonomic nervous system” (ICD-10 diagnostic code: C47.1 for upper extremity and C47.2 for lower extremity) or “malignant neoplasm of other connective and soft tissue” (C49.1 for upper extremity and C49.2 for lower extremity) (Table 1). For patients with more than one claim record with diagnostic codes for extremity STS, the first claim record was counted as the incident time and the patient was counted as an incident case in that year. A total of 3,815 patients with diagnostic codes for extremity STS were identified between 2008 and 2012.

**Table 1:** Diagnostic and procedure codes.

**Diagnostic codes**	
C47.1	Malignant neoplasm of peripheral nerves and autonomic nervous system for upper extremity
C47.2	Malignant neoplasm of peripheral nerves and autonomic nervous system for lower extremity
C49.1	Malignant neoplasm of other connective and soft tissue for upper extremity
C49.2	Malignant neoplasm of other connective and soft tissue for lower extremity
**Procedure codes**	
*Surgery for malignant soft tissue tumors*	
N0232	Removal of soft tissue tumor—malignant
NA282	Limb-salvage procedure for malignant tumor—thigh, scapula, and upper arm
NA283	Limb-salvage procedure for malignant tumor—forearm and lower leg
NA284	Limb-salvage procedure for malignant tumor—hand and foot
S4616	Excision of neuroma—malignant
*Surgery for benign soft tissue tumors* ^a^	
N0233	Removal of soft tissue tumor—subcutaneous benign tumor
N0234	Removal of soft tissue tumor—subfascial or intramuscular benign tumor
S4615	Excision of neuroma—benign
*Radiotherapy*	
HD051~056	Teletherapy
HD057~059	Rotational irradiation
HD061	3-dimentional conformal therapy
HD111, 112	Body stereotactic radiosurgery
*Chemotherapy*	
KK151~156, 158, KK059	Chemotherapic administration

^a^ Only the codes with a claim date within 6 months from the earliest claim date of STS diagnosis at the same location were included.

January 1 to December 31, 2008 was set as a “window period” such that patients were defined as new cases only if their first record of an STS claim occurred after this 1-year period. As most of the follow-up intervals for extremity STS are <1 year [16], we assumed the absence of any claims record with a diagnosis of STS or procedure for STS in the preceding 12 months indicated a new case. Therefore, 1,132 patients with a first claim record in 2008, who may include patients diagnosed before 2008, were excluded. Thus, 2,683 patients were analyzed.

In the HIRA database, the diagnostic codes of STS maybe entered when examinations have been performed only when STS was suspected, with an eventual diagnosis of another entity. Thus, to select genuine extremity STS cases among the 2,683patientswith diagnostic codes for extremity STS, we also investigated if these patients had undergone any specific procedure for STS. The procedure codes for extremity STS included STS-related surgery (i.e., removal of malignant soft tissue tumor [ICD-10 procedure code: N0232], limb-salvage procedure for malignant tumor [NA282 for thigh, scapula, and upper arm; NA 283 for forearm and lower leg; and NA284 for hand and foot], and removal of malignant neurogenic tumor [S4616]), radiotherapy-related procedure (i.e., teletherapy [HD051~056], rotational irradiation [HD057~059], three-dimensional conformal therapy [HD061], body stereotactic radiosurgery [HD111, 112]), and chemotherapy-related procedure (i.e., chemotherapy administration [KK151~156, 158, KK059]). To identify patients who underwent unplanned excision, patients with the procedure codes for the following criteria were also selected: codes for the removal of benign soft tissue tumor for the same location [i.e., N0233, N0234, and S4615] with their claimed date within 6 months prior to the claim date of the diagnostic code for STS. Among the 2,683 patients with a diagnostic code for extremity STS, 1,166 did not have any procedure codes claimed and were thus excluded. Thus, 1,517 patients had claim records for both diagnostic and procedure codes. The diagnostic and procedure codes used for the patient selection process were discussed and selected by a panel of 4 sarcoma specialists including 2 orthopedic oncologists, 1 radiation oncologist, and 1 medical oncologist (Fig 1).

### Data analysis

**Fig 2:**
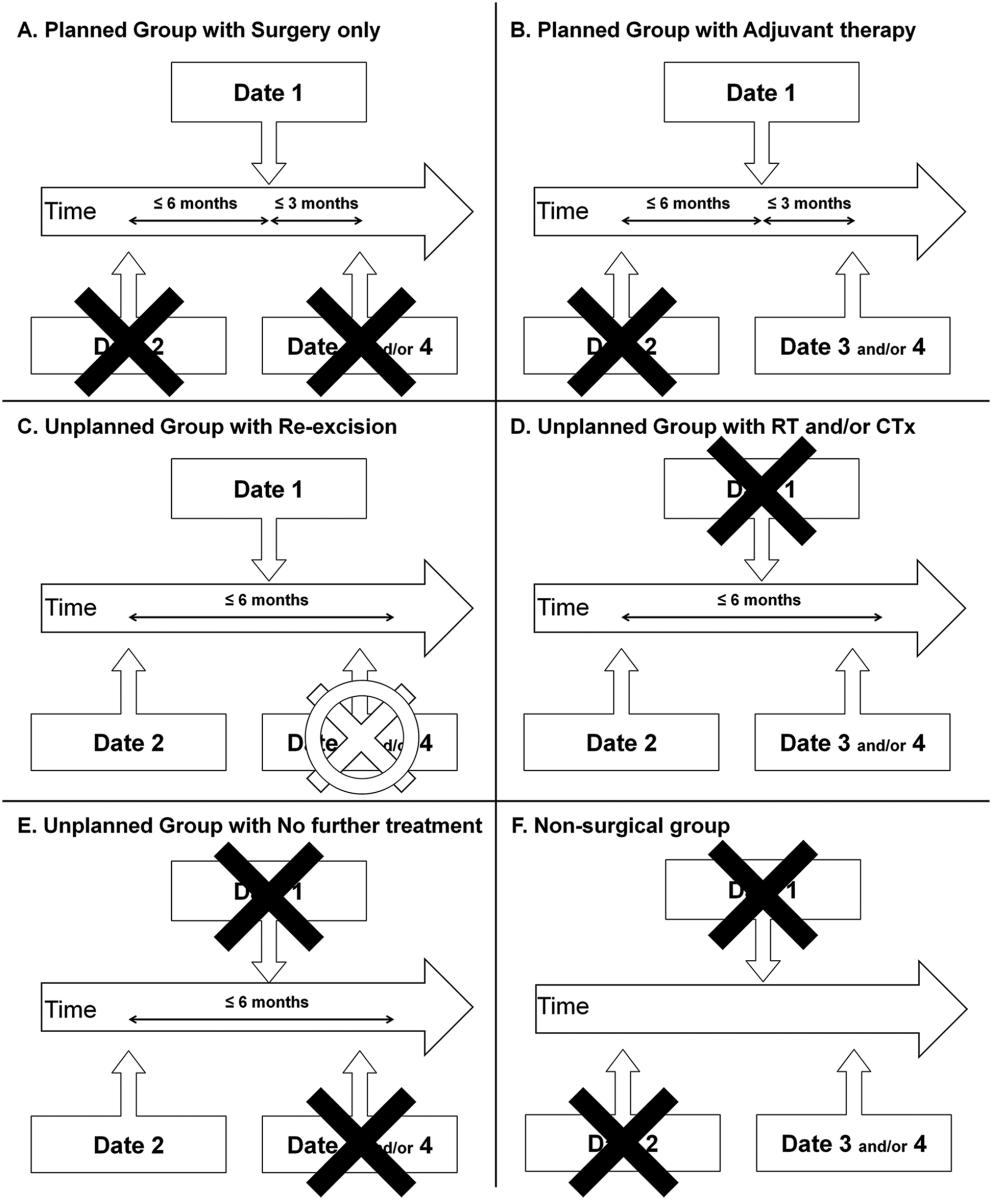
Grouping the patients according to the claim dates for diagnostic/procedure codes. The black symbol of X indicates the need for absence of the specific date for the grouping, and the empty overlapped symbol of O and X means that the presence/absence of the specific date does not affect the grouping. Abbreviations: RT = radiation therapy; CTx = chemotherapy.

To analyze the incidence and treatment patterns of unplanned excision, the patients were divided into 3 groups: (1) those with planned excision (planned group), (2) those with unplanned excision (unplanned group), and (3) those without any surgical treatment (non-surgical group). To divide the patients, we searched for the following claim dates: “date 1” was defined as the claim date of the simultaneous diagnostic and surgical codes for malignant soft tissue tumor; “date 2” was defined as the claim date of the surgical code for benign soft tissue tumor within 6 months from the earliest claim date of STS diagnosis at the same location; “date 3” was defined as the earliest claim date of the procedure code for radiotherapy; and “date 4” was defined as the earliest claim date of the procedure code for chemotherapy. The patients with date 1 and without date 2 were included in the planned group, and those with date 2 regardless of date 1 were included in the unplanned group; meanwhile, patients with neither date 1 nor date 2 (i.e., those who had only date 3 and/or date 4) were included in the non-surgical group (Table 2) (Fig 2)

**Table 2:** Criteria for patient grouping with respect to the treatment pattern of extremity soft tissue sarcoma.

Group	Criteria
Planned group	Date 1 (+) and Date 2 (-)
Unplanned group	Date 2 (+)
Non-surgical group	Date 1 (-) and Date 2 (-)

Date 1: Claim date of the simultaneous diagnostic and procedure codes for malignant soft tissue tumor

Date 2: Claim date of the surgical code for benign soft tissue tumor within 6 months from the earliest claim date of STS diagnosis at the same location

In the planned group (Fig 2A and 2B), the administration of adjuvant therapy was confirmed when the radiotherapy (date 3) or chemotherapy (date 4) within 3 months of date 2 were identified (Fig 2B). In the unplanned group (Fig 2C, 2D and 2E), the performance of re-excision was confirmed when re-excision (date 1) within 6 months after date 2 were identified (Fig 2C). In the subset of patients without re-excision, the administration of radiotherapy or chemotherapy was confirmed when radiotherapy (date 3) or chemotherapy (date 4) within 6 months of date 2 were identified (Fig 2D). For the non-surgical group, we investigated the proportions of palliative radiotherapy (date 3) and palliative chemotherapy (date 4) (Fig 2F).

We also retrieved age, sex, and the anatomical site of the tumor from the claims records. The age when the first diagnostic code of STS was claimed was adopted as the patient’s age, and the anatomical site was classified as upper or lower extremity. The type of hospital where the surgical code for STS or radiotherapy/chemotherapy in the non-surgical group was claimed was recorded. The types of hospitals were classified according to their capacity as defined by the Korean Hospital Association: tertiary hospital, general hospital, hospital, and clinic [17]. The allocation criteria for capacities are as follows: clinic—a medical institution with medical facilities and no difficulty in medical services; hospital—a medical institution with medical facilities and a capacity for more than 30 in-patients; and general hospital—a medical institution with medical facilities, a capacity for more than 100 in-patients, and required specialties such as internal medicine, general surgery, pediatrics, obstetrics and gynecology, radiology, anesthesiology, pathology, psychiatry, and dentistry. Among the general hospitals, a teaching hospital equipped with more specialized facilities and personnel, and treats difficult or rare diseases, is designated as tertiary referral general hospital. Of note, the Korean medical system allows the patients to visit specialists in tertiary hospitals with minimal restrictions [18].

## Results

A total of 1,517 extremity STS patients were identified between 2009 and 2012 (Table 3). The mean age of the patients was 52 years (range, 0.2–98.0 years), and 54% (*n* = 825) were men. Regarding tumor site, 32% (*n* = 480) and 68% (*n* = 1,037) of tumors were in the upper and lower extremities, respectively. The treatment patterns of each group are listed in Table 4. Among the 1,517 patients, 553 (36.5%) underwent unplanned excision. Close to 82% of unplanned excisions were performed in tertiary or general hospitals.

**Table 3:** Demographic characteristics of the patients with extremity soft tissue sarcoma.

	Total	Planned group	Unplanned group	Non-surgical group
Incidence (No. of patients)				
'09	376	191	135	50
'10	393	176	139	78
'11	375	175	142	58
'12	373	194	137	42
'09~'12 (average)	1517 (379.3)	736 (184, 48.5%)	553 (138.3, 36.5%)	228 (57.0, 15.0%)
Gender				
Male	825 (54.4%)	399 (54.2%)	287 (51.9%)	139 (61.0%)
Female	692 (45.6%)	337 (45.8%)	266 (48.1%)	89 (39.0%)
Age, year (range)				
	51.8±19.3 (0.2~98)	53.2±19.1 (0.2~98)	52.1±18.5 (1~90)	46.6±20.7 (0.9~88)
Anatomical location				
Upper extremity	480 (31.6%)	231 (31.4%)	175 (31.6%)	74 (32.5%)
Lower extremity	1037 (68.4%)	505 (68.6%)	378 (68.4%)	154 (67.5%)
Hospital distribution				
Tertiary hospital		463 (62.9%)	278 (50.3%)	185 (81.1%)
General hospital	NA^a^	253 (34.4%)	175 (31.6%)	43 (18.9%)
Hospital		20 (2.7%)	47 (8.5%)	0
Clinic		0	53 (9.6%)	0

^a^ Not applicable because of different definitions for each group, i.e., the distribution of the hospital where the first surgical excision was claimed for the planned/unplanned group and the first radiotherapy/chemotherapy was claimed for non-surgical group.

**Table 4:** Treatment patterns of extremity soft tissue sarcoma.

Planned group (n = 736)		Unplanned group (n = 553)^b^		Non-surgical group (n = 228)	
**Surgery only**	**463 (62.9%)**	**Re-excision**	**240 (43.4%)**	**RT only**	**79 (34.6%)**
**Adjuvant therapy** ^**a**^	**273 (37.1%)**	alone	156 (28.2%)	**CTx only**	**91 (39.9%)**
RT alone	107 (14.5%)	with RT and CTx	25 (4.5%)	**RT and CTx**	**58 (25.4%)**
CTx alone	79 (10.7%)	with RT only	38 (6.9%)		
RT and CTx	87 (11.8%)	with CTx only	21 (3.8%)		
		**RT only**	**23 (4.2%)**		
		**CTx only**	**13 (2.4%)**		
		**RT and CTx**	**15 (2.7%)**		
		**No further treatment**	**262 (47.4%)**		

Abbreviations: RT = radiation therapy; CTx = chemotherapy.

^a^ Adjuvant therapy was defined as radiotherapy or chemotherapy performed within 3 months of the surgery.

^b^ Further treatment in unplanned group was defined as any treatment performed within 6 months after unplanned excision.

**Fig 3:**
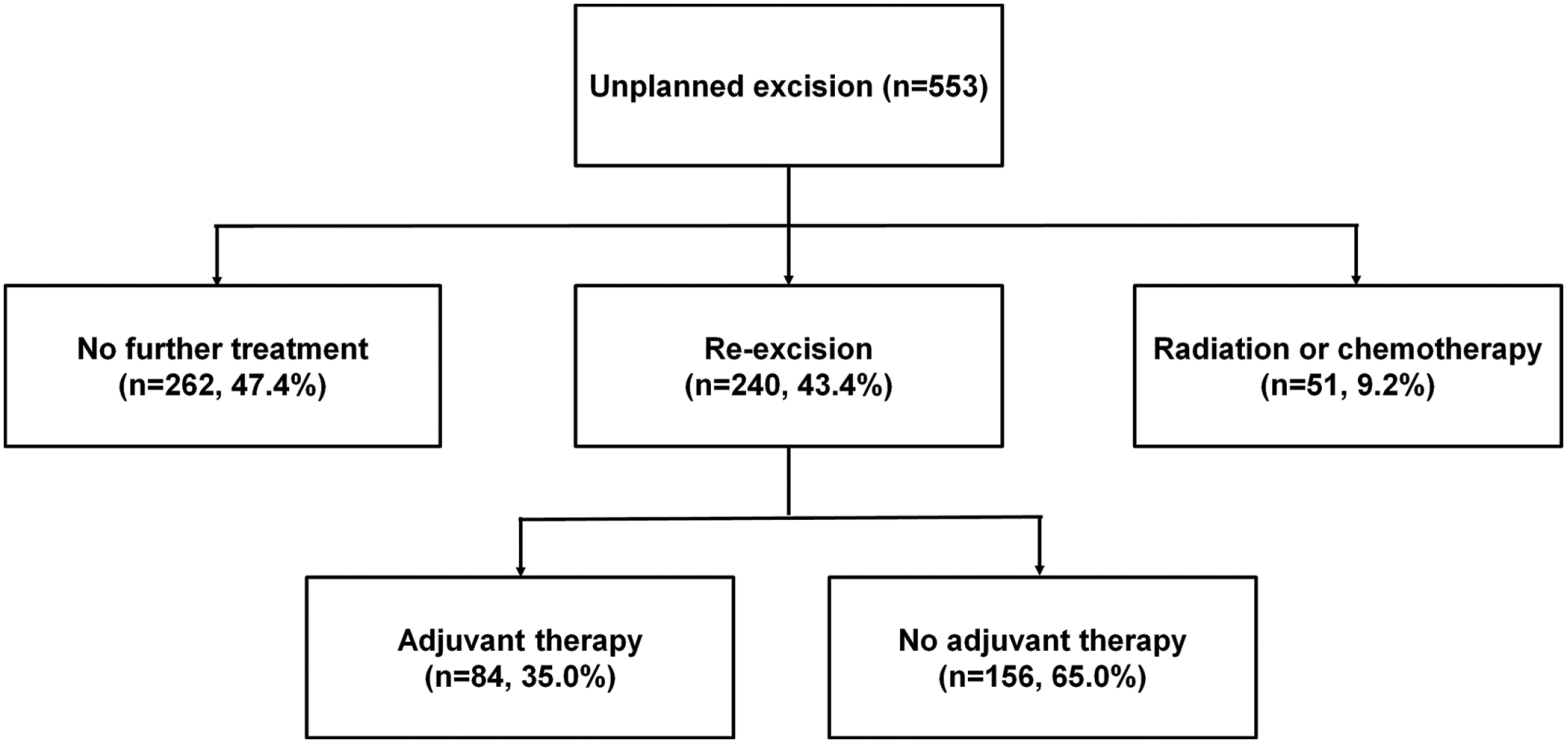
Treatment pattern of extremity soft tissue sarcoma.

Of the 553 patients in the unplanned group, 240 (43.4%) underwent re-excision, 35% of whom (*n* = 84) had radiation therapy and/or chemotherapy with re-excision. Moreover, 51 (9.2%) were treated with radiation therapy and/or chemotherapy without surgery. Furthermore, 47% (*n* = 262) did not undergo any further treatment following unplanned excision (Fig 3).

## Discussion

This study represents the first population-based epidemiologic study of the unplanned excision of extremity STS. An epidemiologic study may provide basic information regarding the incidence and treatment pattern of unplanned excision of extremity STS and help establish preventive or therapeutic measures to reduce the burden of unplanned excision of STS. Therefore, the present nationwide epidemiologic study was performed using the HIRA database, a centralized nationwide healthcare claims registry of Korea. The results of this study provide the evidence that unplanned excision of extremity STS is common in Korea and necessitates the measures to reduce unplanned excisions.

Unique information about the treatment details of extremity STS patients was retrieved from the HIRA database. The procedure codes for cancer are claimed specifically for cancer patients [19]. Moreover, surgeries for extremity soft tissue tumors are coded differently according to the malignancy (i.e., malignant vs. benign) and location of the tumor. By combining the procedure and diagnostic codes from the HIRA database, we generated a nationwide data of the unplanned excision of extremity STS.

Some considerations are needed when interpreting the present results. First, the use of ICD-10 diagnostic codes to identify extremity STS may not accurately estimate the number of incident cases. Although many previous studies used ICD codes to identify STS [20, 21], in the HIRA database, the diagnostic codes for STS may be entered without detailed information on tumor histology. The use of a cancer registry database would have provided more detailed information about the STS such as depth, size, and grade [22]. However, the use of a claims registry rather than a cancer registry was necessary to collect data on treatment to analyze the patterns of treatment for unplanned excision of STS. To circumvent this problem regarding diagnostic inaccuracy, we selected only patients who had both a procedure and a diagnostic code for STS. Previous studies using the HIRA database frequently implemented such a method, which has proven to be both valid and effective [14,23]. Indeed, the present results show a similar incidence of extremity STS as that from the Korea National Cancer Incidence database from the Korean Central Cancer Registry, the nationwide cancer registry of Korea (http://ncc.re.kr/english/index.jsp) [24]. Of note, exclusion of the patients with only procedure codes may have resulted in missing data, such as the patients who did not undergo any treatment although were diagnosed with extremity STS. However, the authors believe that the proportion of such patients is small [24]. STS that did not undergo histologic examination may also be missing in the HIRA system. Masses that are thought to be benign, such as small and/or subcutaneous mass, may not be sent to histologic confirmation. If such a tumor was an STS and is cured after the excision, this tumor would not be registered as an STS. Second, as rather indirect methods were used for identifying unplanned excisions, possible errors exist in estimating the occurrence of an unplanned excision. A possibility exists where an improper surgery is performed but is included as a ‘planned’ excision. To the contrary, an appropriate excisional biopsy may be performed, particularly of a small lesion that is less than 5 cm, but might be coded initially as a benign tumor. Information pertaining to the appropriateness of treatment, such as surgical margin or tumor size, was not available in the HIRA database. Third, as the HIRA database lacks detailed information on variables that decides treatments patterns, such as patient demographics and/or tumor characteristics, the treatment patterns presented in this study may need to be interpreted cautiously. However, this study provides a nationwide profile of the treatment pattern of unplanned excision of extremity STS. Fourth, as this study was based on a database of the Korean population, it may be difficult to generalize the results considering the diversity of medical systems and ethnicities among different countries. Fifth, the validation of the data with actual medical records and pathology reports would be helpful to confirm the results of this study. However, as information from the HIRA database is de-identified, the simultaneous identification of medical records was not possible.

Among all extremity STS patients identified in the present study, 37% underwent unplanned excision. Most reports on the incidence of unplanned excision of STS were hospital based, and we only considered patients who underwent re-excision [7–9,25, 26]. When only the patients who underwent surgical treatment were considered, which was calculated as the number of re-excision cases in the unplanned group (*n* = 240) divided by the sum of the number of cases in the planned group (*n* = 736) and re-excisions (*n* = 240), the proportion of patients with unplanned excision was 25%, which is concordant with previous studies [7–9,25, 26].

Among the patients with unplanned excision, 47% did not undergo any further treatment. In a multi-center hospital-based study of 1,851 patients, one-third of patients who had been referred after unplanned excision did not undergo re-excision [27]. The possible explanations for this observation, which stems from the limited information available from the HIRA database, are as follows. First, it is possible that *en bloc* resections might have been performed in many of these cases, especially in tumors that were superficial and/or less than 5cm in size, which would not have been initially coded as a malignant tumor excision. And no additional re-excision was deemed necessary. In the present study, the majority of hospitals where unplanned excisions were performed were rather higher-level hospitals and. higher-level hospitals tend to have a lower chance of residual tumors at re-excision [28]. Differences in surgical procedures with respect to hospital referral level have also been reported in other cancer types [29–31]. Second, STS for which marginal excisions are not considered as improper treatment, such as well-differentiated liposarcoma in the extremities, might have been included. These tumors would not have been initially coded as a malignant tumor excision and no further treatment may be indicated. In this regard, unplanned excision as defined by the claims codes in this study may not necessarily mean improper treatment. Moreover, well-differentiated liposarcoma can be indistinguishable from benign lipomas on MRI, and could have been coded as benign initially. Third, these cases might have been misclassified because of the inherent nature of claims data, particularly voluntary or involuntary miscoding. However, the number of incident cases of extremity STS during the 4-year study period is concordant with that reported in the annual report based on the Korea National Cancer Incidence Database [10,24].

Close to 80% of unplanned excisions were performed in tertiary or general hospitals. This result is somewhat contradictory to the general notion that unplanned excisions are mostly performed at lower-level referral hospitals, which have much less experience with STS than higher-level referral hospitals. There are several possible explanations for this observation. First, as the lower-level referral hospitals of Korea, namely hospitals and clinics, have limited capacity for surgery, most surgeries on soft tissue tumors are performed in tertiary or general hospitals. Second, as the patients’ access to tertiary or general hospitals is not limited by the medical system, patients with soft tissue tumors may bypass the lower-level hospitals. Third, the use of claims registry database might have overestimated the proportion of tertiary hospitals. A previous hospital-based study about the referral pattern after unplanned excision of extremity STS, 35% of the patients were referred from the tertiary hospitals [32]. Fourth, the general guidelines for the management of extremity soft tissue masses may not have been followed [33].

## Conclusions

This study is the first nationwide population-based study on the unplanned excision of extremity STS. The results provide insight into the incidence and treatment patterns of unplanned excision of extremity STS in Korea. The results may also have implications for establishing preventive or therapeutic measures to reduce the burden of unplanned excision of extremity STS.
